# Evaluating the usability and acceptability of a geographical information system (GIS) prototype to visualise socio-economic and public health data

**DOI:** 10.1186/s12889-021-12072-1

**Published:** 2021-11-24

**Authors:** Joshua Wells, Robert Grant, John Chang, Reem Kayyali

**Affiliations:** 1 School of Life Sciences, Pharmacy and Chemistry, Kingston University London, Penrhyn Road, London, Kingston upon Thames KT1 2EE UK; 2 Faculty of Health, Social Care & Education, Kingston and St George’s, University of London, London, UK; 3 Croydon University Hospital, London, Croydon UK

**Keywords:** Mapping, Public health data, Prevention, Usability, Technology, SUS

## Abstract

**Background:**

Understanding the impact of socio-economic inequality on health outcomes is arguably more relevant than ever before given the global repercussions of Covid-19. With limited resources, innovative methods to track disease, population needs, and current health and social service provision are essential. To best make use of currently available data, there is an increasing reliance on technology. One approach of interest is the implementation and integration of mapping software. This research aimed to determine the usability and acceptability of a methodology for mapping public health data using GIS technology.

**Methods:**

Prototype multi-layered interactive maps were created demonstrating relationships between socio-economic and health data (vaccination and admission rates). A semi-structured interview schedule was developed, including a validated tool known as the System Usability Scale (SUS), which assessed the usability of the mapping model with five stakeholder (SH) groups. Fifteen interviews were conducted across the 5 SH and analysed using content analysis. A Kruskal-Wallis H test was performed to determine any statistically significant difference for the SUS scores across SH. The acceptability of the model was not affected by the individual use of smart technology among SHs.

**Results:**

The mean score from the SUS for the prototype mapping models was 83.17 out of 100, indicating good usability. There was no statistically significant difference in the usability of the maps among SH (*p* = 0.094). Three major themes emerged with respective sub-themes from the interviews including: (1) Barriers to current use of data (2) Design strengths and improvements (3) Multiple benefits and usability of the mapping model.

**Conclusion:**

Irrespective of variations in demographics or use of smart technology amongst interviewees, there was no significant difference in the usability of the model across the stakeholder groups. The average SUS score for a new system is 68. A score of 83.17 was calculated, indicative of a “good” system, as falling within the top 10% of scores. This study has provided a potential digital model for mapping public health data. Furthermore, it demonstrated the need for such a digital solution, as well as its usability and future utilisation avenues among SH.

**Supplementary Information:**

The online version contains supplementary material available at 10.1186/s12889-021-12072-1.

## Background

The case for causality with respect to poor health outcomes is multifactorial. Increasing global mortality as a result of chronic disease continues to challenge health care provision financially [1–3]. Additional contributors include increasing modifiable negative public health behaviours, most notably: smoking, poor diet and lack of physical activity [2, 4]. Despite the growing prevalence of these modifiable risk-factors for long-term conditions, we are seeing an aging population. This is due to improvements in socio-economic status (SES), reductions in birth mortality and a decreasing prevalence of communicable disease [5]. However, these recent advancements in individual status and wellbeing are matched by cumulative morbidity rates and a seemingly unachievable demand for healthcare resources, further fuelling the growing burden on national care provision [6, 7]. Even though individuals may have benefitted from changes in SES, this is not a nationwide phenomenon. The literature suggests those with a lower SES are increasingly likely to be exposed to more unhealthy behaviours and outcomes compared to their more affluent counterparts [8].

From a socio-economic perspective, measures such as the Townsend Score [9] or Index of Multiple Deprivation [10] (IMD) are examples of tools that attempt to correlate SES to overall wellbeing through a number of factors including employment, education, health deprivation and disability. Such tools frequently utilise infographics or images to better convey information given the public’s familiarity with this form of data visualisation. The aim of such an approach is to enhance understanding, engagement, and interest in the topic. Evidence of this application already exist within academia [11–13] and research [14, 15], but in an era of globalisation, multi-media and increasing access to technology, the role of visualisation continues to expand. Internationally, there is a wealth of literature discussing the use of visualisation tools in relation to public health data. A systematic review produced in 2014 [16] identified various themes that impact implementation of visualisation tools. A total of 88 documents across 5 bibliographic databases met the inclusion criteria with a focus on epidemiological data of infectious disease. A key observation from the review was that the potential to provide an abundance of relevant information was offset by poor interpretation of data. Lack of follow up in terms of tool usability plans and data dissemination were reported as contributing factors to a decreased uptake in this approach to data analysis. Furthermore, service-users were found to have different needs, lack of support and a general misunderstanding of how visualisation tools can be utilised [16].

A more positive demonstration of Geographical Information System (GIS) implementation in public health was undertaken by St James’s Hospital in Dublin, assessing population Vitamin D levels in the local catchment area [17]. The study visually demonstrated the relationship between seasonal changes, gender, and age in the variability of Vitamin D concentrations. This approach supported the concept of service provision based on identifying high risk areas for example, lower Vitamin D levels in men vs women living in the same area. From this observation, male patients could be the target group for service provision in specified localities. Similarly, Curtis et al. [18] produced evidence depicting regional trends in diabetes prevalence contrasted with available regional resources. This resulted in data analysis that helped categorise “high risk – low resource (HRLR)” populations. The capacity of visualisation to highlight HRLR areas or populations has value, providing an additional layer of evidence for evaluating and identifying correlations between current health inequalities and available services [19, 20].

A systematic review by Luan and Law [21] highlights the utilisation of web-based GIS public health surveillance. The authors describe variations in functionality across several tools such as Google Maps, OpenCalais and ArcGIS to name but a few. Elements perceived as integral for overall functionality include interactivity and usability, irrespective of an individual’s technical background or experience with GIS. Furthermore, the authors emphasise that translation of raw health data via GIS should produce easily interpretable results which can be effectively communicated. Other notable examples include Mapbox, Open Layers and GIS Cloud, however the choice is often determined by user preference and desired functionality.

As part of a collaborative funded project with Public Health Croydon (PHC) and Croydon University Hospital (CUH), the researchers were tasked to create an accessible mapping model to demonstrate variations in socio-economic deprivation across the Borough of Croydon. Upon completion, PHC and CUH assigned the research team with local authority and hospital data to evaluate two case studies based on local needs using the prototype mapping model. Further work was conducted to assess the usability of the model with users outside of the public health domain, which centred on identifying the perceptions of a range of stakeholders (SHs) in the use of interactive visualisation models of public health data. In addition, the study explored the potential uses and value of data visualisation techniques, barriers and challenges to data use and access as well as currently employed methods.

## Methods

### Synopsis

The study consisted of two phases. Phase 1 included designing an accessible and interactive mapping model. Post-completion, the model was presented to PHC and CUH where further case studies were requested and evaluated based on local needs, specifically to explore paediatric vaccination uptake and early readmissions within the Borough. The second phase implemented a quantitative and qualitative questionnaire as part of one-to-one interviews with SHs to gauge the perceived usability and acceptability of the model. The aim of collating SH perceptions was to support proof of concept with respect to validating the model for visualisation of public health data without specialist intervention. Ethical approval for this study was provided on 30/01/2017 by the Kingston University Ethics Committee (1213/045). Methods were reported according to the Consolidated Criteria for Reporting Qualitative Research (COREQ) [22].

### Setting & study population

Five distinct SH groups were identified for this study including: GIS experts, healthcare professionals (HCPs), public health commissioners, voluntary sector workers (VS) and members of the public. Recruitment was undertaken across South-London using a convenience and snowballing strategy [23] to ensure that sufficient SHs could be identified for the study. The study aimed to recruit enough SHs to achieve adequate content saturation and identify themes throughout the interview process. Data utilised for the purpose of developing the mapping model were provided by CUH and PHC.

### Map design

**Fig. 1:**
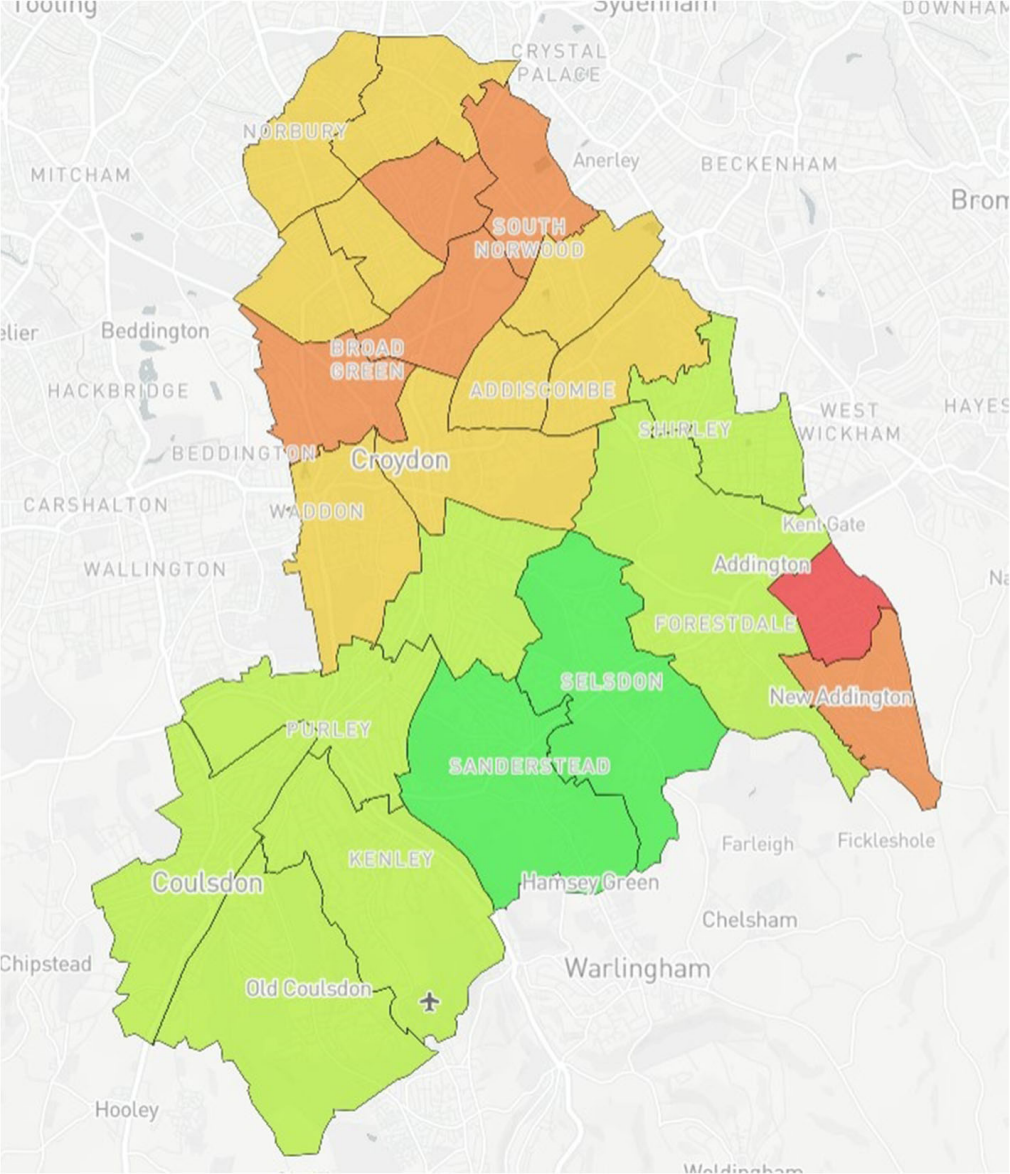
Index of Multiple Deprivation (IMD)^a^ by ward in Croydon. ^a^Green (least deprived), Red (most deprived)

**Fig. 2:**
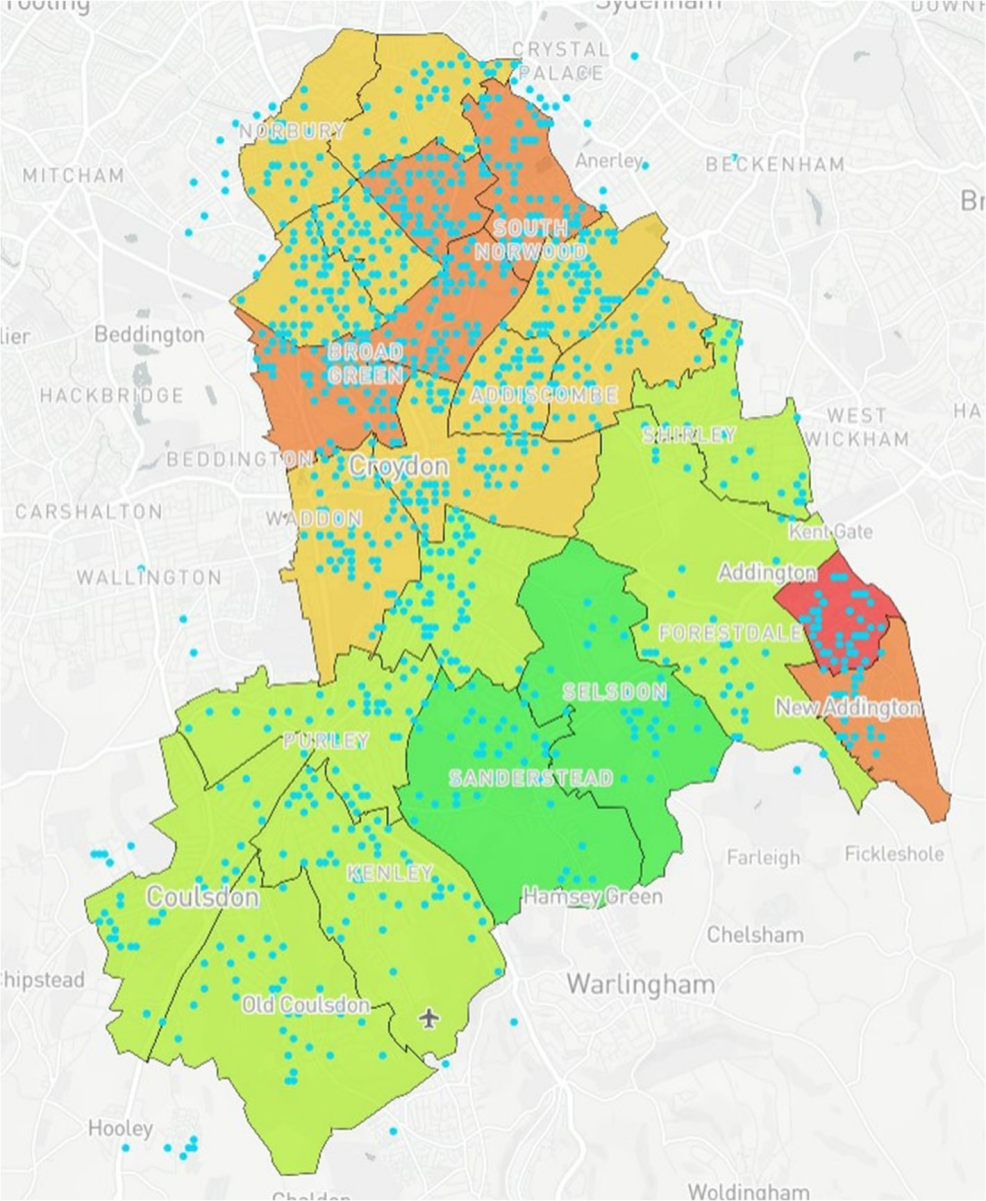
Anonymised addresses^a^ of children aged 5 that have received an MMR vaccination. ^a^Blue dots reflect truncated long/lat values equivalent to the address

**Fig. 3:**
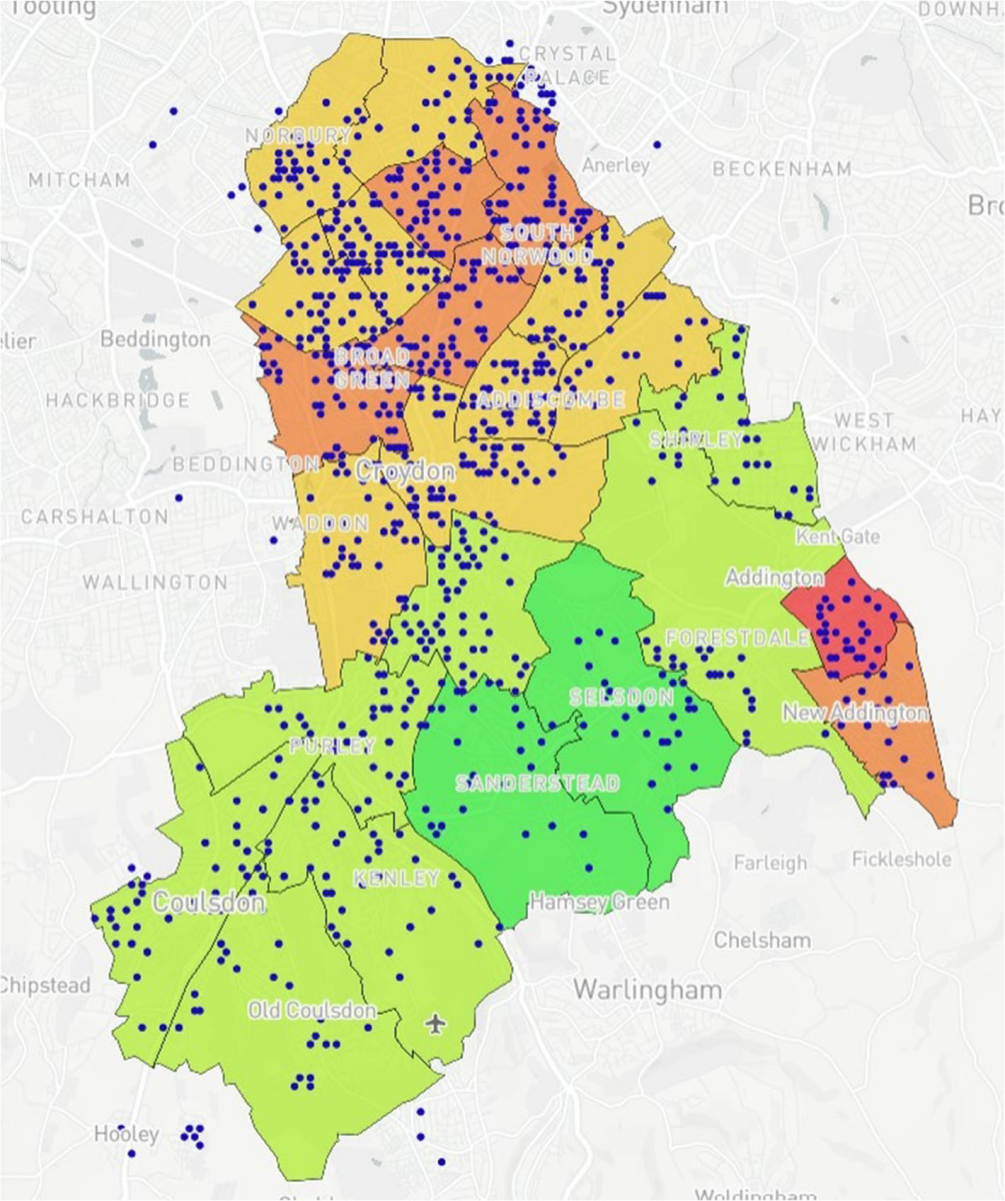
Anonymised addresses^a^ of children aged 2 that have received an MMR vaccination. Blue dots reflect truncated long/lat values equivalent to the address

**Fig. 4:**
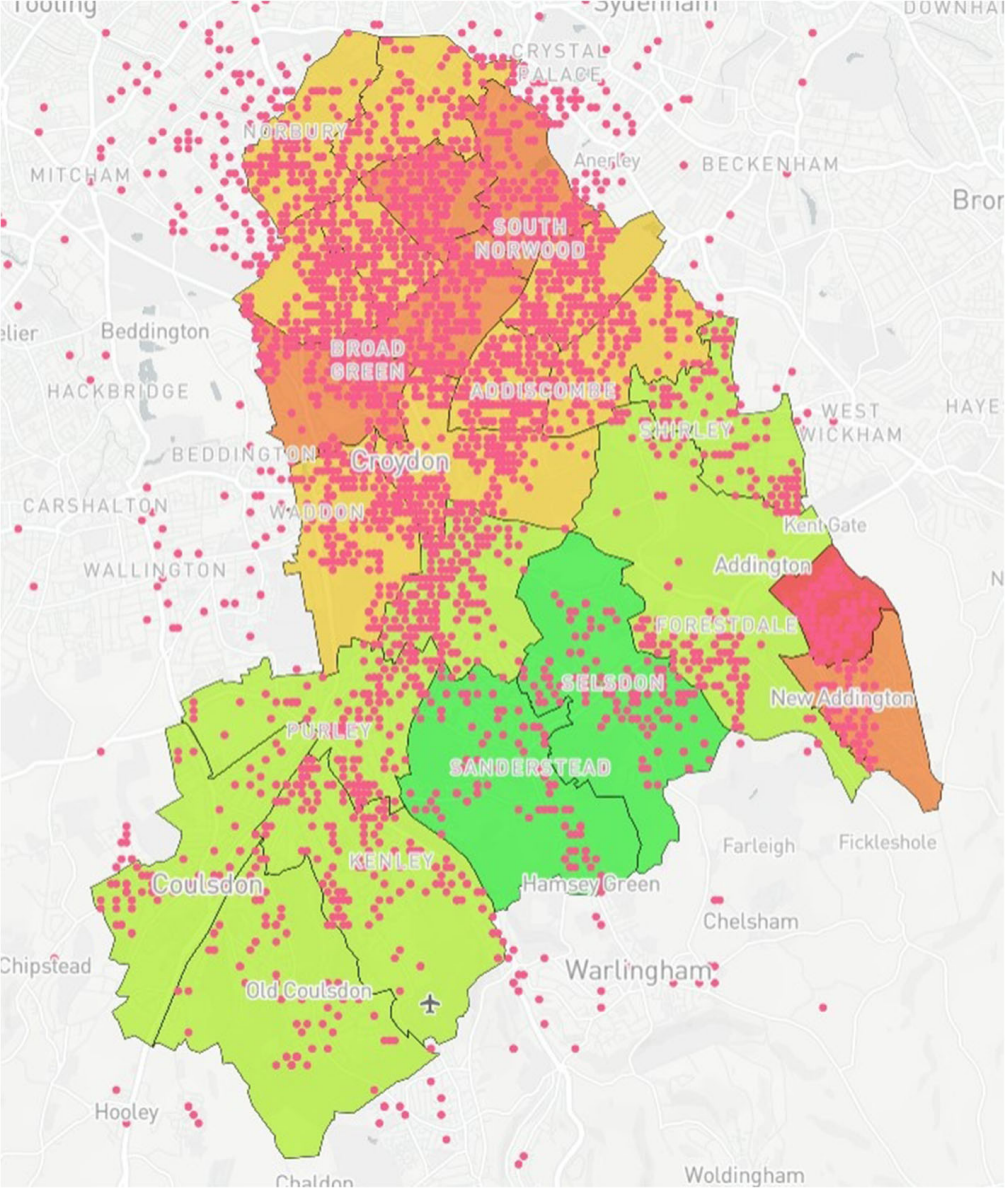
Anonymised addresses^a^ of registered births in Croydon from 2015 to 2016 ^a^Pink dots reflect truncated long/lat values equivalent to the address

**Fig. 5:**
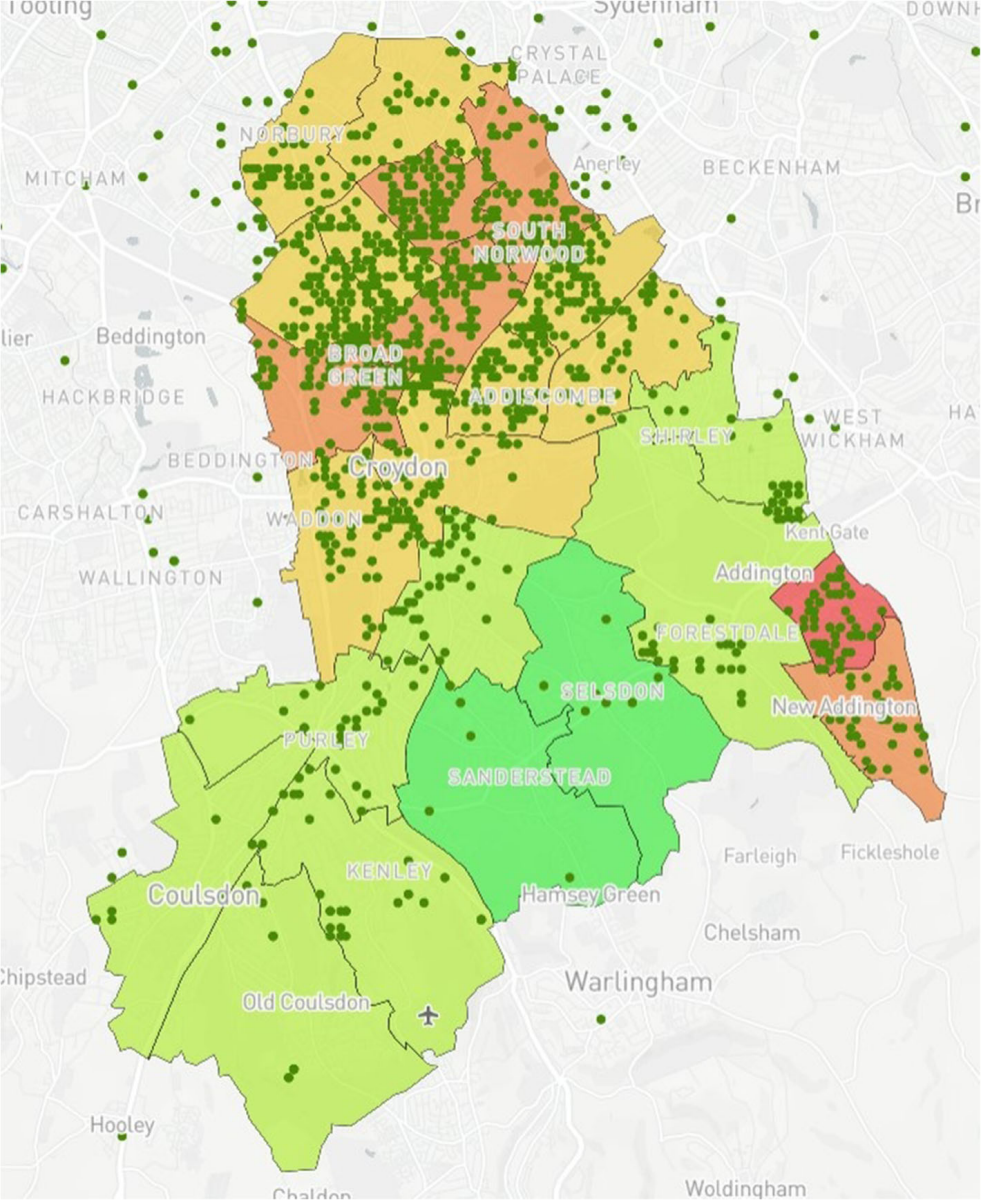
Anonymised addresses^a^ of registered births in Croydon by ethnicity (Black African) from 2015 to 16. ^a^Green dots reflect truncated long/lat values equivalent to the address

**Fig. 6:**
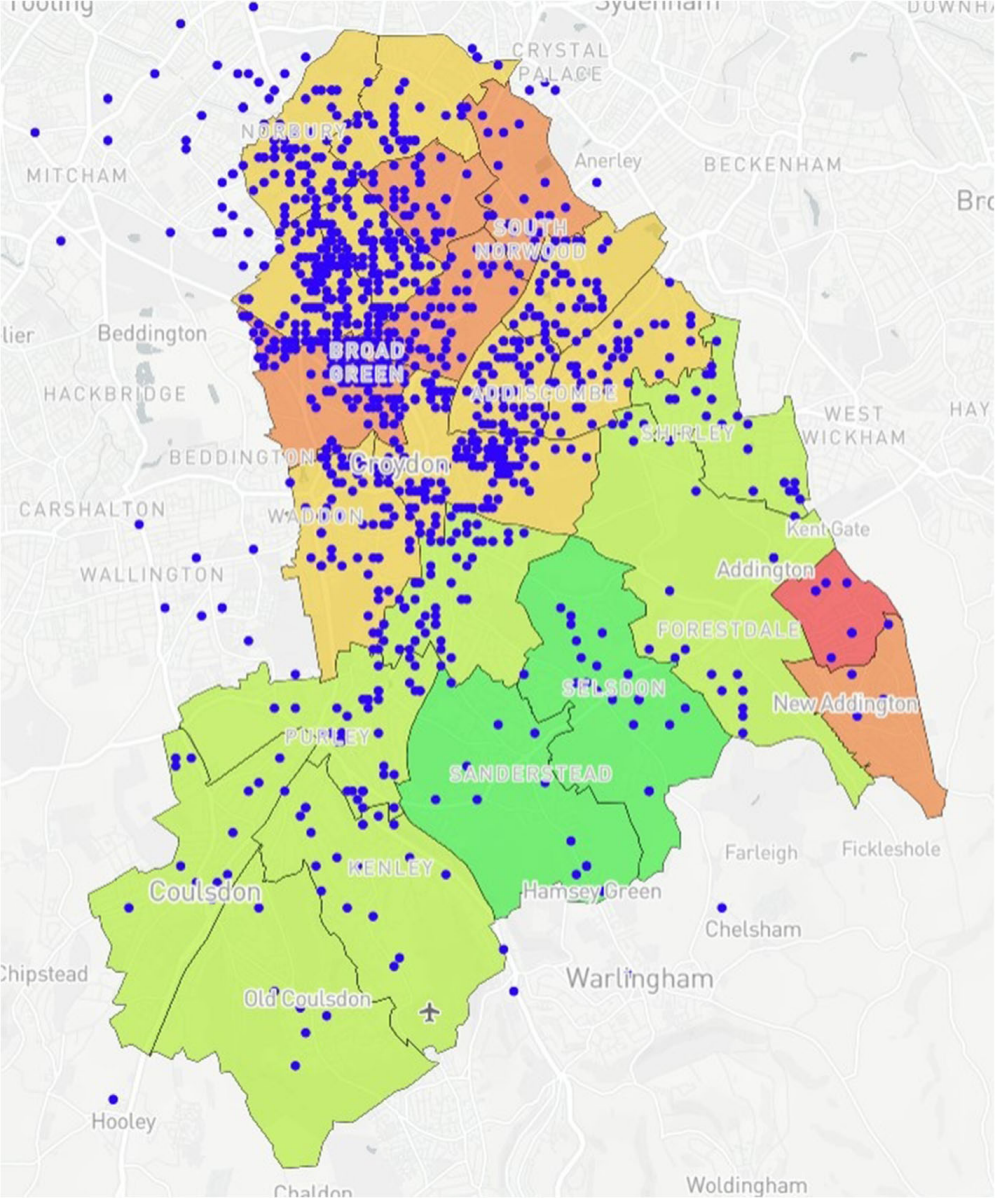
Anonymised addresses^a^ of registered births in Croydon by ethnicity (Asian) from 2015 to 16. ^a^Blue dots reflect truncated long/lat values equivalent to the address

**Fig. 7:**
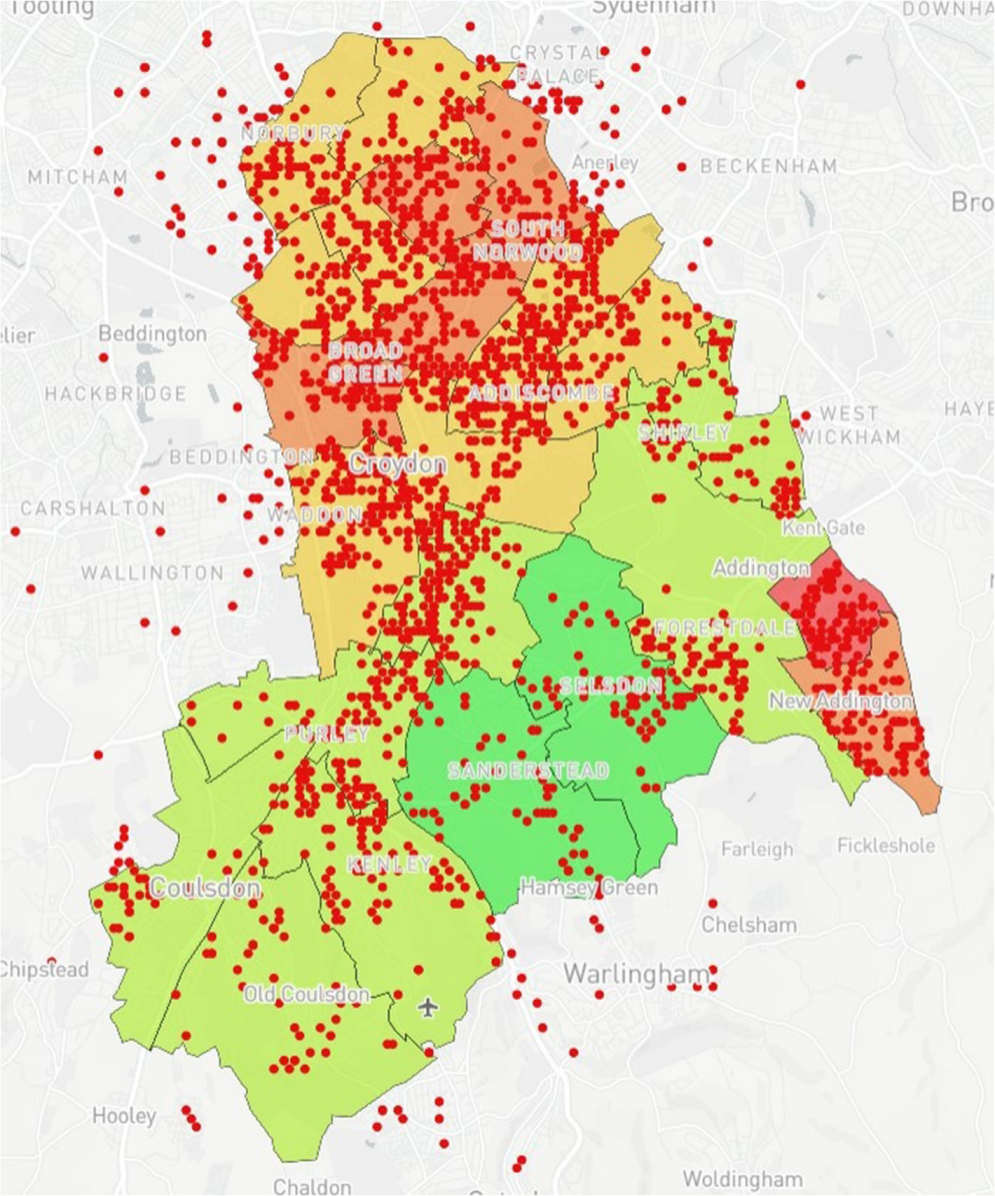
Anonymised addresses^a^ of registered births in Croydon by ethnicity (White European) from 2015 to 16. ^a^Red dots reflect truncated long/lat values equivalent to the address

**Fig. 8:**
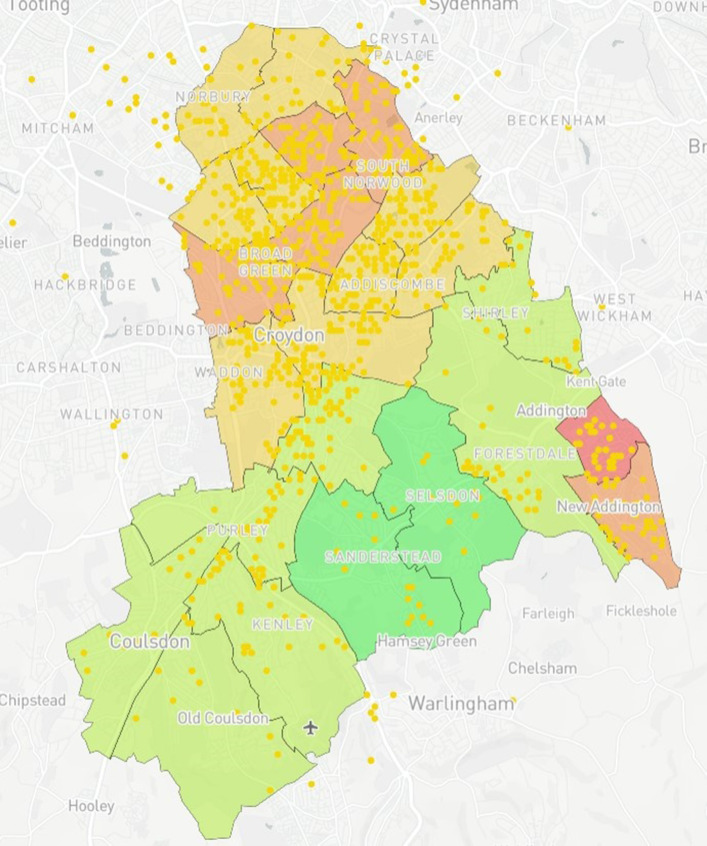
Anonymised addresses^a^ of registered births in Croydon by ethnicity (Mixed/Other) from 2015 to 16. ^a^Yellow dots reflect truncated long/lat values equivalent to the address

**Fig. 9:**
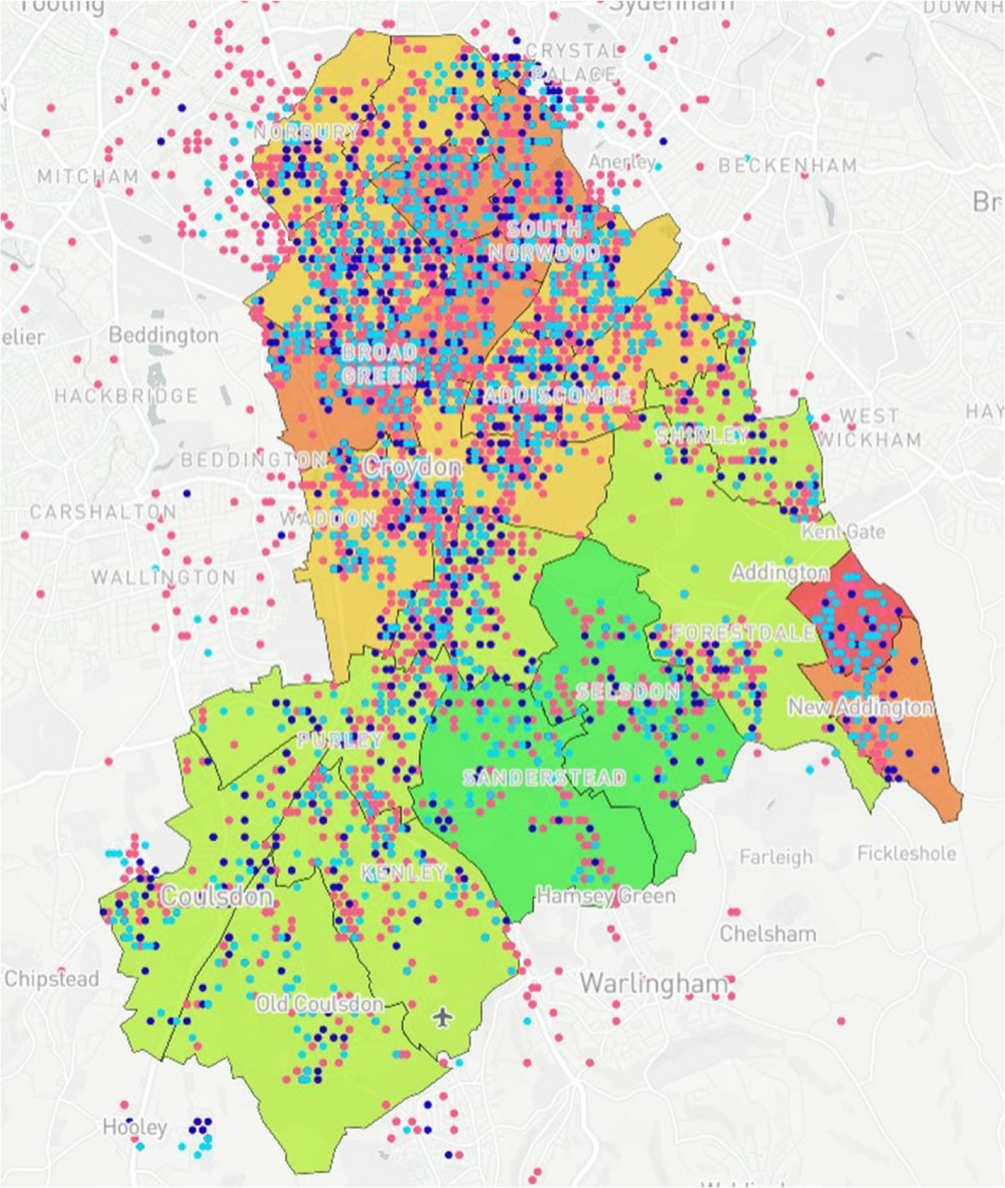
Case Study 1 - Total Births^a^, MMR Aged 2^b^ & MMR Aged 5^c. a^Total Births (Pink); ^b^MMR Aged 2 (Dark Blue); ^c^MMR Aged 5 (Light Blue)

**Fig. 10:**
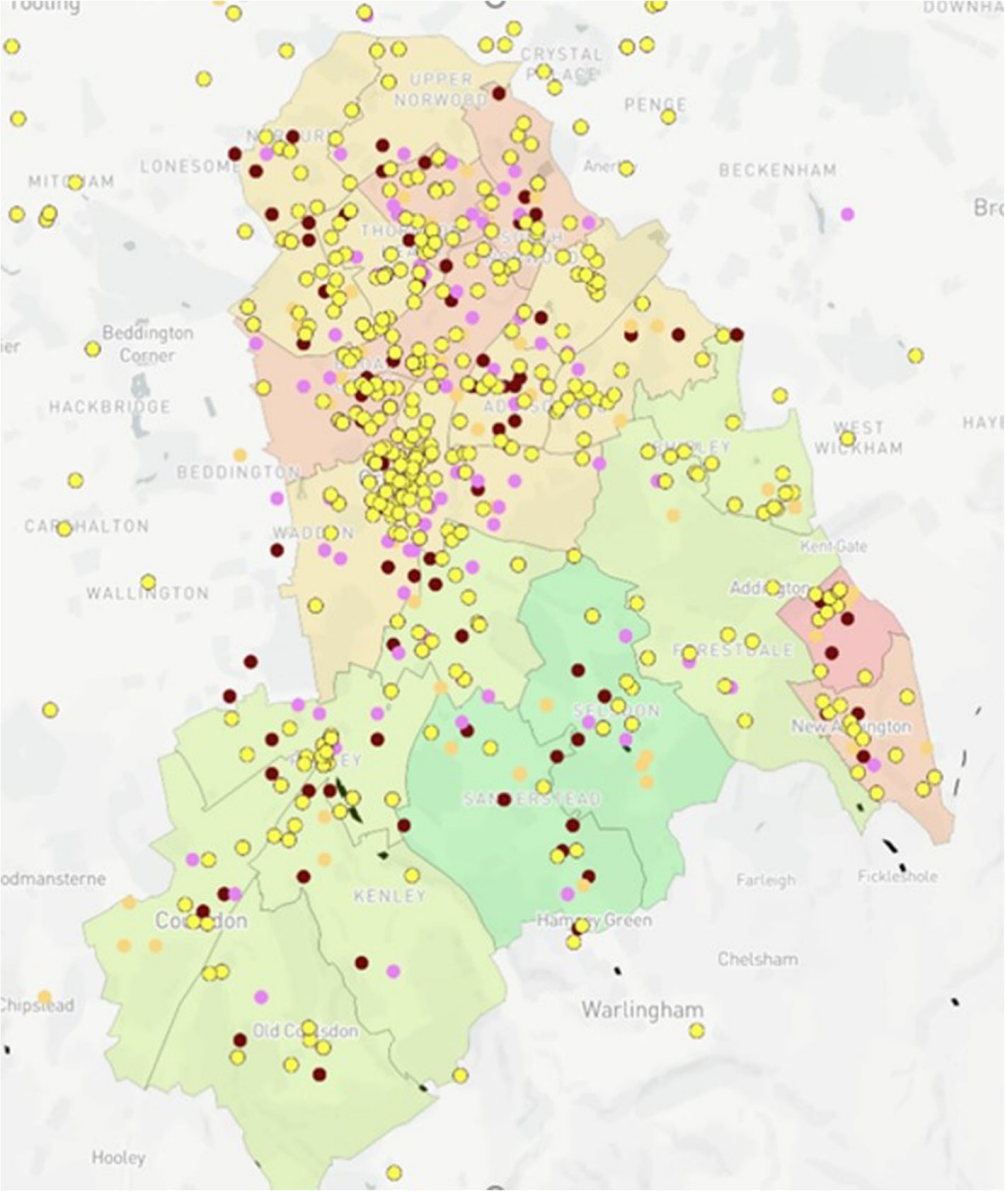
Case Study 2 - Readmission^a^ vs Local Organisations^b a^Congestive Heart Failure (Pink), Rheumatoid Arthritis (Red), Falls (Orange)^. b^Civil Service Organisations (Yellow)^.^ NB – 19 conditions can be added to the map but were excluded from the image in order to demonstrate clarity. Additionally, the locations of community pharmacies and GP surgeries were an additional functionality but have been excluded from the image for clarity

**Fig. 11:**
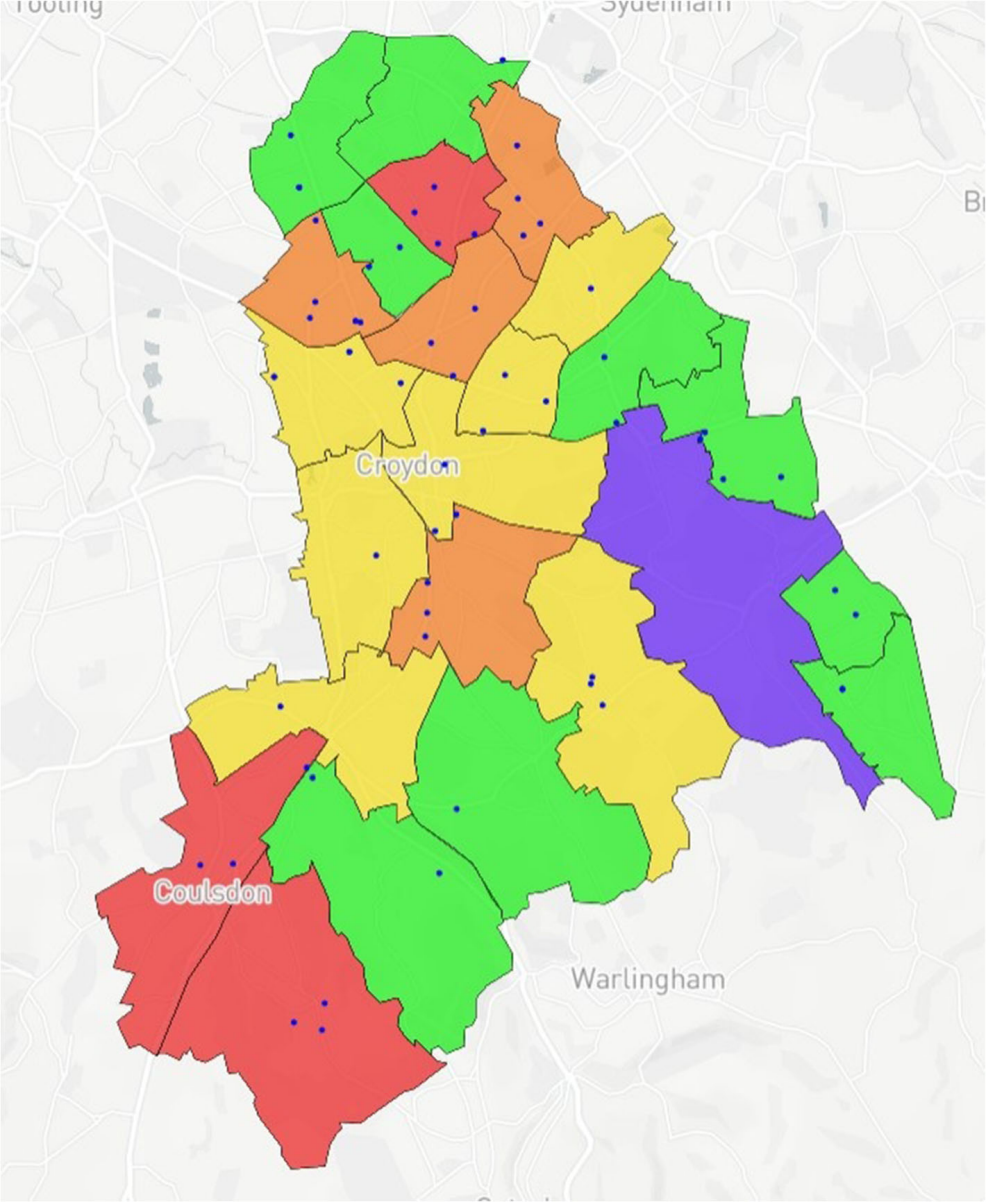
Case Study 3 - MMR Vaccination uptake by ward including GP Surgery location. Blue = No data. Green = < 10 children who have not been vaccinated. Yellow = < 20 children who have not been vaccinated. Orange = < 30 children who have not been vaccinated. Red = > 30 children who have not been vaccinated. Blue Dots = GP Surgery locations

The prototype was created using Mapbox [24]. Mapbox was chosen for this study given its capacity to produce complex maps without the need for a strong technical background while integrating interactive elements and custom base layers. Furthermore, Mapbox was free for use within the study, which included open-access online tutorials to support map development. The map is a multi-layered model demonstrating wards with their corresponding IMD values within Croydon (Fig. 1). IMD values were acquired through census data provided via GOV.UK [25]. Upon completion, the prototype was presented to PHC and CUH who then provided data for two additional case studies. For Case Study 1, using data provided by CUH, 10 additional data layers (Figs. 2, 3, 4, 5, 6, 7, 8, 9, 10, 11) were compiled and overlaid onto the mapping model (Table 1). Additional zooming and panning functions were included as part of the model. A protocol to design the mapping model has previously been reported [26].

**Table 1:** Mapping Model Data Layers

Layer Number	Information Displayed	Figure Location
1	Wards with corresponding IMD values (Borough of Croydon)	Figure 1
2	Anonymised addresses of children aged 5 who received a Mumps, Measles & Rubella (MMR) vaccination	Figure 2
3	Anonymised addresses of children aged 2 who received an MMR vaccination	Figure 3
4	Anonymised addresses of registered births 2015–16	Figure 4
5	Anonymised addresses of registered births 2015–16 by ethnicity: Black African	Figure 5
6	Anonymised addresses of registered births 2015–16 by ethnicity: Asian	Figure 6
7	Anonymised addresses of registered births 2015–16 by ethnicity: White European	Figure 7
8	Anonymised addresses of registered births 2015–16 by ethnicity: Mixed/Other	Figure 8
9	Total births versus MMR vaccination for children age 2 and 5	Figure 9
10	Readmission rates versus local organisation in Croydon	Figure 10
11	MMR vaccination by ward versus locations of GP surgeries	Figure 11

Ward IMD values were grouped as follows: < 10, > 10/< 20, > 20/< 30, > 30/< 40, > 40. The five groups were categorised and given corresponding colour codes so that the groups would be visible on the map with green being the least deprived and red the most deprived (See Fig. 1).

For Case Study 2, CUH provided longitudinal data from a 12-month observational study [27] highlighting the primary diagnoses for patients readmitted to the Trust within 30 days of their original discharge. The data were used to create an overlay for the mapping model (Fig. 10), highlighting those conditions for which there were over 100 readmissions during the study period versus ward IMD values and local civil services.

Case Study 3 aimed to further develop the concept of case study 1, streamlining data visualisation for vaccination rates across Croydon. The location of GP surgeries in the Borough of Croydon were added as an overlay. The proportion of two- and five-year olds who had not been vaccinated vs the overall paediatric population age two and five who were eligible for MMR vaccination by ward, was calculated. The results of this calculation allowed the researcher to contrast the proportion of unvaccinated children by ward vs the local distribution of GP surgeries and deprivation level by ward (Fig. 11).

### Case study analysis

The data provided to develop each model had not previously been analysed by PHC or CUH. Two researchers were tasked with independently reviewing the models to determine whether visualisation could be used to assess significant findings. The researchers were asked to view the maps and write down any findings for each case study, the results were consolidated and analysed using qualitative content analysis before SH engagement to validate findings prior to Phase 2 interpretation of the models.

### Evaluating the usability and acceptability of the map design

Phase 2 of the study consisted of interactive semi-structured interviews (See Additional Files 1, 2, 3, 4, 5) with individual SHs where they were given the opportunity to use models reflecting data from case studies 1–3. Interviews were carried out by the lead researcher who was registered on the MPharm programme at their institution at the time of the study. The interviewer identifies as male; however, all participants were met by both the researcher and project lead who, identifies as female, prior to consenting to the interview where any concerns could be discussed. No issues were disclosed, and all participants agreed to interview individually with the lead researcher. The lead researcher had previous experience in clinical interviews with patients as part of the MPharm programme. Prospective participants were provided with information in advance via email regarding the purpose of the study (research as part of MPharm programme), credentials of the interviewer and scope of the interview.

Usability was examined through the qualitative examination of perceived ease of use (PEOU) and perceived usefulness (PU) of the mapping models [28]. Interview questions were designed to capture SH feedback as a reflection of overall acceptance of the model e.g., Likes/dislikes, satisfaction, suggested uses for the maps and overall benefits associated with the data visualisation model.

To supplement the qualitative element of the interview, participants were given the opportunity to use the interactive case study maps followed by completion of a validated quantitative Likert scale tool, known as the System Usability Scale [29] (SUS). SUS is frequently utilised in the assessment of novel technology models. The scale produces a numerical value that is indicative of the SHs’ perception of the system in terms of PEOU, satisfaction and confidence among other factors. Dependent on the statement, Likert scores are either subtracted from 5 or have 1 subtracted from their value. The final score is then multiplied by a factor of 2.5 to achieve an overall score out of 100. (See Additional File 6).

The study was intended to demonstrate the usability of a mapping model that could be developed with little to no previous experience in data visualisation and hence the chosen lead researcher was not a usability specialist or data visualisation expert. A semi-structured interview approach in addition to the SUS was chosen above methods such as heuristic evaluation or cognitive walkthroughs to evaluate usability given that these methods often require the input of those with specialist experience to guide the assessment process [30]. Furthermore, the use of semi-structured interviews and content analysis allowed the end-users and researcher to utilise natural language as a method to assess usability, avoiding the need for expert input, additional learning by end-users or potential ambiguity in analysing results associated with other structured query language (SQL) such as graphical and visual languages [31]. The open-ended interview approach aimed to generate discussion not only on the usability of the tool but also general themes of data utilisation, visualisation and personal experience.

Interviews took approximately 20–30 min and were audio recorded following provision of consent and then transcribed verbatim. Field notes made during the interview were not included in the transcript. Data from each interview including the transcript, SUS and smart technology surveys were pseudo-anonymised prior to being uploaded onto either the relevant Word or Excel document for data analysis. The transcriptions were examined by the lead researcher and project lead using qualitative content analysis [32], whereby they were categorised to highlight major themes with corresponding sub-themes. Saturation was achieved at interview 11, therefore 4 interviews provided supplementary data to support the identified themes. The interview scores for the SUS were analysed in Excel Version 16 [33] and compared using a Kruskal-Wallis H Test. A *p-*value was calculated to examine any difference in usability across the five SH groups with values < 0.05 being considered as statistically significant.

## Results

### Case study analysis

Case Study 1 –Both researchers identified a disparity in distribution of paediatric populations by ethnicity vs ward level of deprivation across the Borough of Croydon. Participants registered as White European (Fig. 7) populated predominantly affluent wards vs those registered as Asian (Fig. 6) or Black African (Fig. 5) being more densely populated in wards with higher levels of deprivation, particularly in the North of the Borough

Case Study 2 – The researchers identified a greater distribution of civil services within more populated areas of the Borough (Fig. 10). Although not an unexpected finding, this did highlight the potential for social prescribing across the Borough, particularly given the higher number of readmissions in the most densely populated wards which experience the most significant levels of deprivation

Case Study 3 – No significant correlation between the distribution of GP surgeries vs vaccination uptake was observed by either researcher. However, the researchers identified a correlation between ward deprivation level and vaccination uptake across the Borough (Fig. 11) using the simplified approach to data visualisation. Interestingly, uptake was poorer in the most affluent wards across Croydon, particularly in the South of the Borough, which were less densely populated

### Interactive interviews

In total, 15 interviews were completed throughout the course of the study, with a minimum of 2 representatives of each SH. Demographics are presented in Table 2. The modal age fell into the 30–39 age category with the modal level of education being a Master’s degree or equivalent (*n* = 6, 40%).

**Table 2:** Demographic characteristics of study participants

	Demographic Characteristics (***n*** = 15)
	No. SH interviewed per category
Commissioner	2
Expert	2
Healthcare Professional	3
Public	5
Voluntary Sector	3
**Age**	
< 18	0
18–29	3
30–39	5
40–49	2
50–59	3
60–69	1
70–79	1
≥ 80	0
**Highest Level of Education**	
No formal education	0
GCSE/O-Level or Equivalent	1
A-Level or Equivalent	1
Bachelors or Equivalent	5
Masters or Equivalent	6
Doctoral Degree or Equivalent	2
**No. participants that own ‘smart’ technology**	
Yes	14
No	1

Overall, 93% (*n* = 14) owned a smart device. The one participant who did not own a smart device did not complete the smart technology survey. Over 50% of participants indicated that they carried out four of the six activities multiple times a day (Table 3). Overall, the use of smart technology amongst the participants was deemed to be frequent, with no suggestion that there would be any case of significant digital illiteracy among survey participants. When comparing frequency of smart technology use and perceived usability of the mapping model, no significant association was seen.

**Table 3:** Frequency of Smart Technology Use

	Activity (***n***,%)					
Frequency	Send a text	Make a call	Send/check emails	Use social media	Download an app	Use an app
**Multiple times a day**	13, 93%	7, 50%	13, 93%	8, 57%	–	12, 86%
**Once a day**	1, 7%	7, 50%	1, 7%	2, 14%	–	1, 7%
**Once a week**	–	–	–	–	5, 36%	–
**Once a month**	–	–	–	–	1, 7%	1, 7%
**Less than once a month**	–	–	–	1, 7%	8, 57%	–
**Never**	–	–	–	3, 21%	–	–

Through content analysis of the interviews, three major themes were identified during the study:

1. Barriers to Current Use of Data
2. Design Strengths & Improvements
3. Multiple Benefits & Usability

These three themes broadly cover the results from the interviews. Each major theme has been categorised based on a series of sub-themes that are included as part of the sections mentioned above.

### Barriers to current use of data

#### Sub-Themes:

##### Accessibility is the greatest barrier to current use of data

Two-thirds (66%, *n* = 10) of participants stated that inaccessibility to data, both professionally and personally, was the biggest barrier they faced. Issues with access ranged from not having the required permissions due to workplace restrictions (Expert, Commissioner and HCPs), lack of digital literacy e.g., not knowing how to search for information (VS) and too much information or spam/adverts (Public).

*“There is also a technology thing. There’s an issue with accessing things online with the council laptop. I can’t even get a look at it [webpage] to see if it’s worth my while” –* Expert 2.

*“If you look at it [labels] for face value, you might not know what they were” –* Public 2.

### Design strengths & improvements

#### Sub-Themes:

##### Interactivity and overlaying improve overall functionality and usability

After exposure to the models, over half (60%, *n* = 9) of participants specified that the interactive feature played a key role in aiding usability. The interactive elements including the sliders, zoom and pan function were deemed as important mechanisms in aiding the use of the mapping model by 87% (*n* = 13) of participants. SHs reported on average that they would strongly agree that the model was easy to use and would agree that the functions were well integrated.

“*It’s interactive. The presentation is really clear and it’s obvious to see stuff. It’s great to see that there are different options to choose how much, and what you can and can’t see*” - Public 2.

*“Yeah I think the sliders, the zooming, were great. I like the way you can use them to clear the data”* – VS 2.

##### The models enabled users to visualise, understand & identify correlations in case study data

Overall, 60% (*n* = 9) of those interviewed described the value of using a visual model to understand data sets. Roughly half (53%, *n* = 8) provided specific examples of how the mapping model could correlate data such as birth ethnicity with deprivation, and the accessibility of local services and GP surgeries with vaccination rates as well as the distribution of chronic disease at a ward level within the Borough, hence highlighting areas of potential high risk.

*“It shows you how the African population is mainly in the deprived areas … I would imagine European [births] would be pretty much everywhere (turns up slider) oh yeah there they are!”* – VS 3.

*“You can visualise the colour changes which means you can spot the high-risk areas much easier” -* HCP 1.

##### Design and data improvements for the mapping model

The main general improvement suggested was the addition of a key (60%, *n* = 9). Over a quarter (27%, *n* = 4) discussed changing the colour scheme to aid visualisation, particularly for those with visual impairment in mind. SH specific changes ranged from the addition of local school data to compare vaccination uptake and additional functionality of a distance scale to support comprehension of the mapping model in terms of zooming and panning while observing the data in each case study.

*“You could have a zoom function at the bottom to show how zoomed in I am or a distance function to show how many kilometres”* – HCP 2.

*“Decisions around to take vaccinations or not, the information that is spread through media is reinforced by conversations had in school by parents such as about MMR, it would be interesting to look at uptake from different schools and whether it correlates”-* Commissioner 2.

##### The model was easy to use

Overall, 80% (*n* = 12) of participants indicated that the model provided a quick, easy or clear visual demonstration of data. Participants strongly agreed that the model was easy to use and time efficient.

*“It just makes it easier doesn’t it? You can just see the areas where there is a problem can’t you … rather than having to look at loads and loads of data especially when people don’t have a lot of time”* – HCP 2.

### Multiple benefits & usability

#### Sub-Themes:

##### Numerous applications exist for the mapping model

Personal applications varied between SHs and included: Providing information about localities to support activities such as purchasing a house, finding impromptu community activities, and identifying community assets e.g., art galleries. Professional applications included: Informing decision making with respect to the provision of health services such as monitoring the spread of disease vs immunisation and tracking pollutants or particulate matter and their environmental impact. Furthermore, one example included identifying differences in GP services provided locally to help compare community outcomes. Both experts felt that the mapping model would be cost effective, avoiding the need for software licenses or analysts. In total, 40% (*n* = 2) of the public participants suggested the model had personal value for activities such as assessing area house pricing with relation to crime statistics. All VS SHs (100%, *n* = 3) felt the model was valuable as an interface between other organisations. It was noted that both commissioners (100%, *n* = 2) thought the model would be valuable in mapping clinical service provision such as immunisation distribution as demonstrated by Case Study 3. Two (66%) of HCPs felt the value of the model centred on clinical service provision mapping and population needs as demonstrated with Case Study 2 when investigating contributors to secondary care readmission in Croydon.

*“It would be interesting to see what public health services are available in the local area”* – Healthcare Professional 3.

*“I’d like to use it [the map] to show data that correlates to buying a house in the area – crime rates, house prices, a key with that information on”* – Public 5.

*“Focusing on something like diabetes, or musculoskeletal [disease] … the map can present [data] in terms of demographics, availability and trends around service usage as well”* – Commissioner 2.

*“I can see it as a health planning resource … for instance you could track the outbreak of disease and spread in the community … [another] one would be the military … For pollution and the environment I can see if being useful, you can map particulate matter in the atmosphere or pollutants and track those changes”* – HCP 1.

*“I’d like to see variation in GP service provision, this way you can challenge poor outcomes”* – Commissioner 1.

##### The model is a tool for generating discussion of outcomes

Of those interviewed, 40% (*n* = 6) specifically described the model having utility as a conversational tool for professional discussions. These included discussing public health outcomes with stakeholders such as councils (Experts) and as a method to identify high-risk low resource areas which can support resource provision planning (Commissioners). All VS SHs (100%, *n* = 3) stated the model could be used to communicate either metrics with funders or as a tool whereby the user could answer questions by inputting their own data.

*“Being able to share with stakeholders easily … particularly being able to fiddle with it [the map] yourself … what we’re often trying to do is communicate with someone else so you want the most simple method possible to show that and to save time”* – Expert 1.

*“It could be that those GPs are working in higher deprivation areas … if there’s a factor that’s impacting MMR uptake … actually maybe you need more resources in that area to target that and so you can use that [the map] to have a discussion about how best to do that”* – Commissioner 1.

*“[Funders] don’t really understand what we’re delivering and it can be difficult to explain … I’d like [the map] to be able to have input from our database … you could see [data] in a context against the rest of Croydon and compare” –* VS *3.*

### System usability scale

All participants (*n* = 15) completed the SUS survey as part of the interactive interviews to investigate the perceived usability of the model. A mean SUS score of 83.17 was calculated, as well as the individual mean statement scores for the survey (Table 4).

**Table 4:** Results from the SUS survey conducted during the interactive interviews

Participant ID	SUS Total Score^**a**^	Statement 1^**b**^	Statement 2	Statement 3	Statement 4	Statement 5	Statement 6	Statement 7	Statement 8	Statement 9	Statement 10
VS 3	100	5	1	5	1	5	1	5	1	5	1
HCP 3	100	5	1	5	1	5	1	5	1	5	1
Expert 1	95	5	1	5	1	3	1	5	1	5	1
Expert 2	95	5	1	5	1	3	1	5	1	5	1
HCP 1	92.5	5	1	5	2	5	2	5	2	5	1
VS 2	90	5	2	5	2	4	1	5	1	5	2
Public 4	90	4	1	5	2	5	1	4	1	4	1
Public 3	87.5	4	1	5	2	5	1	4	1	4	2
Public 5	87.5	4	2	4	2	5	1	4	1	5	1
HCP 2	77.5	3	1	5	2	4	3	5	2	4	2
Public 1	75	4	1	4	2	4	2	5	2	4	4
Public 2	70	4	2	4	2	4	2	4	2	4	4
VS 1	70	4	3	5	5	3	1	5	2	4	2
Commissioner 1	70	2	1	4	1	3	2	4	3	4	2
Commissioner 2	47.5	2	2	4	5	3	3	4	3	3	4
**Mean Scores**	**83.17**	**4.07**	**1.40**	**4.67**	**2.07**	**4.07**	**1.53**	**4.60**	**1.60**	**4.40**	**1.93**

^a^Total SUS score calculated out of 100 using the tool scoring methodology described in ‘Data Collection & Analysis’

^b^Statements for the SUS Survey: 1) I think that I would like to use this system frequently;2) I found the system unnecessarily complex;3) I thought the system was easy to use;4) I think I would need the support of a technical personal to be able to use this system;5) I found the various functions in the system were well integrated; 6) I thought there was too much inconsistency in this system;7) I would imagine that most people would learn to use this system very quickly; 8) I found the system very cumbersome to use;9) I felt very confident using this system; 10) I needed to learn a lot before I could get going with this system

Table 4 - Results from the SUS survey conducted during the interactive interviews.

A Kruskal-Wallis H Test was performed to determine any difference in usability based on SH SUS scores. The result was found to be non-significant (*p* = 0.094). This demonstrated no significant difference in perceived usability of the mapping model between SH groups.

## Discussion

As part of a collaborative initiative with PHC and CUH, this study has developed an interactive GIS methodology based on local needs that can deliver visualised public health data while identifying its usability and applications across different SH groups. This study also explored the influence of socio-demographics and smart technology use on the acceptability of the mapping model.

The literature suggests that the average SUS score for a system is 68 [29]. However, beyond a score of 68 exists ‘above average’ percentile rankings for systems on graded criteria. The overall SUS score for the model in this study was 83.17. This score is indicative of a ‘B’ grade in terms of usability, falling within the top 10% of scores (> 80.3%). The literature suggests that a ranking of > 80.3% not only defines a system as ‘good’ in terms of usability, but also demonstrates an increased likelihood of users recommending the technological system to a friend [29].

The results are consistent with other methods that assess acceptability such as the Technology Acceptance Model (TAM) [34]. This method identifies factors such as PEOU and perceived usefulness PU as the most important in determining the likelihood of a new technology being adopted. This study demonstrated that participants identified numerous applications for the mapping model. This result validated the PU aspect of the model assessment in accordance with TAM [34]. PEOU as a concept was also established through the quantitative SUS measure, with participants strongly agreeing the model was easy to use. This finding was also apparent through reported participant confidence with the model. The mapping model was able to provide a succinct summary of various public health data sets through visualisation of both a 12-month observational study (readmission diagnosis) and retrospective data analysis (paediatric vaccination rates), with participants reporting ease of use as the most positive SUS outcome. These results support the concept of mapping as a tool for information provision by improving understanding and satisfaction in concurrence with the literature [35].

The case studies were specifically designed and analysed in response to local needs identified by leads across PHC and CUH with further testing conducted to examine the usability and acceptability of the model with SHs not working within public health. With respect to overall distribution of MMR vaccinations for children aged two & five, there is a relatively even spread of recorded vaccinations across the Borough, with greater reporting in more densely populated areas as would be expected. However, when compared to the demographically marked births, there is an apparent disparity with those recorded as White European being registered in more affluent areas versus those registered as either Black African or Asian births in less affluent areas. With the apparent population diversity and greater exposure to deprivation for non-White European persons, it was expected that overall vaccination rate by ward would closely mirror the reports from the IMD for Croydon. Surprisingly, some of the best vaccination reports are from the most deprived wards in the North and East of the Borough, whereas those with the lowest deprivation reported some of the worst vaccination uptake for children aged 2 and 5. Case study 3 also shows the distribution of GP surgeries, with a greater number concentrated in the north of the Borough, however this mainly accounts for the greater population density. A 2016 US study [36] found a significant relationship between paediatric MMR vaccination and affluence, with those from a more affluent background demonstrating lower vaccination uptake. The authors note the trend to decline vaccination based on personal belief is fairly novel. However, despite the unexpected result produced by the mapping model, no full conclusion can be made between IMD, vaccination uptake and ethnicity within the scope of this study. It may be that those living in the UK in more affluent areas may prefer to vaccinate their children privately, and/or outside of the Borough. Nevertheless, these results were of great interest to study participants who found they could deduce these types of correlations using the interactive elements of the model.

Manovich and McInerny et al [37, 38] discuss the positive impact of interactivity on interpreting information displayed by visual data models. This evidence supports the themes identified throughout the study, with the SHs suggesting the most important contribution to usability of the mapping model being the interactive component. The “overlaying” function also supplemented SH’s ability to interpret data displayed by the mapping model. Tippett [39] highlights the value of this visual and constructivist approach e.g., manipulating data layers on the map, as a process through which data comprehension is enhanced. As a result, improvements to usability and the adoption of technology are also observed, as demonstrated by this study through PEOU as a factor of the TAM. Furthermore, the study provides evidence that the interactive functions of the map were well integrated through analysis of the SUS scores.

Interestingly, despite an overall mean SUS score of 83.17, some disparity was noted between participants with a range of 52.5. The lowest SUS score provided was 47.5, with the next closest score being 70. An examination of causal relationships between the result and the demographics for the participant identified one potential implicating factor, the participant’s age. The lowest scoring participant (SUS Score = 47.5) fell into the 60–69 age category. The literature indicates a lower level of acceptability and adoption of smart technologies above the age of 60, and hence this may have been a factor impacting the usability of the mapping model for the participant [40]. However, in contrast the eldest participant in the 70–79 age category provided a SUS score of 87.5. Additionally, this was also the only participant in the study to not own any smart technology. Although this finding relates to a small sample (*n* = 2), it demonstrates that age as a demographic is not always an exclusive indicator of technology acceptance. Chung et al. [40] support this conclusion in a cross-sectional population TAM study of 248 participants which found no statistical significance between age and PEOU. It may be the case that the participant who provided a score of 47.5 also felt they lacked some understanding or confidence in answering the SUS statements, however this was not concordant with the attitudes of other SHs in answering the SUS survey. Furthermore, no correlation between frequency of smart technology use or demographics and SUS scores was seen in this study, a finding in agreement with the current literature [40].

The study aimed to determine the desired purpose for the mapping model based on SH views. As previously discussed, numerous applications were proposed by the SHs during the interviews. Overall, the mapping model was identified as a powerful tool in enabling the visualisation of public health data, as demonstrated by the case studies. SHs described the value of the mapping model as a tool for generating discussion with respect to outcomes and population needs. SHs suggested the purpose of the model could be as a tool for planning the provision of health services or identifying already locally available services. This suggestion mirrors previous NHS strategy, referred to as Sustainability and Transformation Plans (STP), which aimed to highlight the needs of the local population to better streamline service delivery. Rummery [41] emphasises the need for a methodology linking data and outcome; this study has provided evidence that SHs recognise the map as an appropriate model for this function. SHs recognise the map as a tool that enables users to identify correlations in data, specifically generating conversation regarding visual trends. This functionality to visualise trends in data is another approach to quantifying the impact of factors on public health similarly to IMD, as well as other examples of GIS technology use discussed in the literature [16–18, 42]. Marmot et al [43] emphasise the implications of growing health inequality in the UK. Therefore, tackling the impact of SES disparity on health will be both a challenge and a priority in the future of public health service provision.

One correlation of interest to participants, deduced from case study 2, was the abundance of civil service organisations dispersed across Croydon. A range of support organisations were mapped including those targeting alcohol and substance misuse, disability and specialist epilepsy care for young people. Case study 2 demonstrated how GP surgeries and pharmacies are greatly outnumbered by civil service organisations with 819 recorded at the time of this study. In the advent of social prescribing, the researchers question whether such civil organisations would be suited to support patients post-discharge to alleviate the pressures on primary care. Evidence suggests that SES has a significant impact on readmission, hence integrating health and social care when discharge planning may prove to be beneficial [44, 45]. However, numerous barriers including a lack of infrastructure, poor implementation and a lack of funding persist [46]. Developing methods, such as the mapping model, may serve to alleviate some of these pressure through the utilisation, streamlining and distribution of data among SHs when service planning, such as in case study 2.

This study has demonstrated the usability of an accessible and low-resource intensive mapping model as a method of data visualisation. More work is needed to determine the impact of such a model in areas such as commissioning and social prescribing. Limitations for the use of data visualisation can be categorised into three main areas including poor interpretation of data, lack of understanding for the role of this methodology as well as accessibility, as described by Caroll et al [16]. This study attempted to offset this by including a diverse study population with five distinct SH groups, and an interactive mapping model element to support data interpretation across the three case studies. Additional focus groups to examine user-experience may have benefitted the analysis of the model’s usability and acceptability, hence this was a limitation for the study. This study had a small recruitment sample, therefore quantitative results should be treated cautiously with further work required, however content analysis identified saturation of themes throughout the interviews.

## Conclusions

This study has provided evidence to suggest that the mapping model, which has low requirements for both resources and expertise, is both usable and acceptable among different groups of SHs using validated qualitative and quantitative methods. PEOU and the integration of interactive elements were demonstrated as important factors in determining the usability of the model. Numerous applications and benefits of utilising this method of data visualisation both within, and beyond the public health sector were identified by SHs reflecting positive PU among participants. Further work should look to examine the usability of the model using other comprehensive methods such as heuristics or cognitive walkthroughs. In addition, evaluation of the utility and usability of the model should be explored through user-experience to contextualise future findings. In conclusion, this research has identified challenges facing the provision of public health such as the widening gap in health equality as well as integration of health and social care. This model may be of particular use as a mapping methodology in the current pandemic given the developing body of evidence that has identified disparities in health outcomes due to socio-economic inequality.

## Supplementary Information

**Additional file 1.** Interview guide for commissioner participants

**Additional file 2.** Interview guide for voluntary sector participants

**Additional file 3.** Interview guide for expert participants

**Additional file 4.** Interview guide for healthcare professional participants

**Additional file 5.** Interview guide for public participants

**Additional file 6.** Interview guide for generic interactive session

## Data Availability

The database used for generation of the mapping models includes confidential information regarding the addresses of children in Croydon which have received an MMR vaccination. The data also includes registered births within the Borough and details of those patients who were readmitted to Croydon University Hospital, hence public access. Specific precautions were taken in order to anonymise all data included as part of the visual mapping models. These data would not be available from a public repository and nor can it be made available as specific administrative permission was provided to the lead researcher who had an established honorary contract with Croydon University Hospital in order to carry out the research. All supplementary data, such as ward boundaries and census data, have been cited in the article and an active link to the data sets have been provided in the references, as well as below. IMD values were accessed via gov.uk using a publicly open database: https://www.gov.uk/government/statistics/english-indices-of-deprivation-2015. Ward boundaries were accessed via Mapshaper using a publicly open database: https://mapshaper.org/

## Abbreviations

CUH: Croydon University Hospital
GIS: Geographical Information System
GP: General Practitioner
HCP: Healthcare Professional
HRLR: High-Risk Low-Resource
IMD: Index of Multiple Deprivation
MMR: Mumps, Measles and Rubella
PEOU: Perceived Ease of USE
PHC: Public Health Croydon
PU: Perceived Usefulness
SES: Socioeconomic Status
SH: Stakeholder
STP: Sustainability and Transformation Plans
SUS: System Usability Scales
TAM: Technology Acceptance Model
UK: United Kingdom
US: United States
VS: Voluntary Sector
