# Supplementary material for: Evaluating the usability and acceptability of a geographical information system (GIS) prototype to visualise socio-economic and public health data
Source: BMC Public Health. 2021 Nov 24;21:2151. doi: 10.1186/s12889-021-12072-1 (PMC8611402; doi:10.1186/s12889-021-12072-1)
Supplement: Supplementary file 3 — Additional file 3. Interview guide for expert participants [file 12889_2021_12072_MOESM3_ESM.docx]

ID:

Date:

Demographic Questions

1. Do you currently own any ‘smart’ technology e.g. ‘smart’ phone
   1. Yes
   2. No
2. If answered “Yes” to question 1, please answer the following question. How often do you undertake the following activities?

|  | Send a text | Make a phone call | Send/check emails | Use social media | Download an app | Use an app on my device |
| --- | --- | --- | --- | --- | --- | --- |
| Multiple times in the day |  |  |  |  |  |  |
| Once a day |  |  |  |  |  |  |
| Once a week |  |  |  |  |  |  |
| Once a month |  |  |  |  |  |  |
| Less than once a month |  |  |  |  |  |  |
| Never |  |  |  |  |  |  |

1. What is your highest level of education?
   1. No formal education
   2. GCSE/O-Level or equivalent
   3. A-Level or equivalent
   4. Bachelors Degree or equivalent
   5. Masters Degree or equivalent
   6. Doctoral Degree
2. Are you currently working?
   1. Yes
   2. No
3. If answered “Yes” to question 4, please provide your job title:
4. What age category are you in?
   1. Less than 18 years old
   2. 18 – 29
   3. 30 – 39
   4. 40 – 49
   5. 50 – 59
   6. 60 – 69
   7. 70- 79
   8. 80 years of age or older
5. Tell me about your role:
6. What data do you use in your role? (format, source, type, presentation, demographic, public health )
7. What barriers impact your use/access of data? (Too much data, blurry images, unclear topic, ethics, clinical governance)
8. If applicable, please provide an example of a data mapping method you have used previously:
9. In terms of your role, what data would you find helpful? (format, source, presentation, demographic, maps, numerical/verbal)
10. In your opinion, what do you think would be the benefit of using this method of data visualisation?
11. Question asking participant to interpret data from Case Study 1
12. Question asking participant to interpret data from Case Study 2
13. Question asking participant to interpret data from Case Study 3
14. What did you like about the model? (presentation, format, ease of understanding, type of map)
15. What did you dislike about the model? ( format, presentation, type of information, lack of understanding)
16. What would you like to see on the model? (Type of data, change in layout, user specific, different display, different device.)
17. What would you use this type of data mapping for?
18. Any other comments?

Ask the participant to complete the SUS questionnaire.

End of interview.
