# Supplementary material for: Evaluating the usability and acceptability of a geographical information system (GIS) prototype to visualise socio-economic and public health data
Source: BMC Public Health. 2021 Nov 24;21:2151. doi: 10.1186/s12889-021-12072-1 (PMC8611402; doi:10.1186/s12889-021-12072-1)
Supplement: Supplementary file 5 — Additional file 5. Interview guide for public participants [file 12889_2021_12072_MOESM5_ESM.docx]

ID:

Date:

Demographic Questions

1. Do you currently own any ‘smart’ technology e.g. ‘smart’ phone?
   1. Yes
   2. No
2. If answered “Yes” to question 1, please answer the following question. How often do you undertake the following activities?

|  | Send a text | Make a phone call | Send/check emails | Use social media | Download an app | Use an app on my device |
| --- | --- | --- | --- | --- | --- | --- |
| Multiple times in the day |  |  |  |  |  |  |
| Once a day |  |  |  |  |  |  |
| Once a week |  |  |  |  |  |  |
| Once a month |  |  |  |  |  |  |
| Less than once a month |  |  |  |  |  |  |
| Never |  |  |  |  |  |  |

1. What is your highest level of education?
   1. No formal education
   2. GCSE/O-Level or equivalent
   3. A-Level or equivalent
   4. Bachelors Degree or equivalent
   5. Masters Degree or equivalent
   6. Doctoral Degree
2. Are you currently working?
   1. Yes
   2. No
3. If answered “Yes” to question 4, please provide your job title:
4. What age category are you in? (Please tick the relevant box)
   1. Less than 18 years old
   2. 18 – 29
   3. 30 – 39
   4. 40 – 49
   5. 50 – 59
   6. 60 – 69
   7. 70- 79
   8. 80 years of age or older
5. If you were to use the internet, what information/type of information would you be looking for? (google map, transport, shop opening hours, food/drink, online shopping)
6. What is the most common type of information you search for?( google map, transport, shop opening hours, food/drink, online shopping)
7. How would you usually access this type/any type of information? (phone, tablet, computer, apps)
8. What type of information do you think you are missing? (format, source, type of data e.g. local services, lack of accessibility, not up to date)
9. Are you happy with the presentation and why/what would you change? (format, source, ease of access, site of access, device availability e.g. phone app or not)
10. What do you find difficult about using/accessing information? (lack of access, incorrect information/not updated, no available information)
11. What would make using/accessing this information easier? (an app, improved presentation, formatting, increased support of lack of technical ability)
12. What is the most common type of transport you use?
13. Do you suffer from any chronic conditions, and if so which conditions? (No obligation to answer this question)
14. Question asking participant to interpret data from Case Study 1
15. Question asking participant to interpret data from Case Study 2
16. Question asking participant to interpret data from Case Study 3
17. What did you like about the model? (presentation, format, ease of understanding, type of map)
18. What did you dislike about the model? ( format, presentation, type of information, lack of understanding)
19. What would you like to see on the model? (Type of data, change in layout, user specific, different display, different device.)
20. What would you use this type of data mapping for?
21. Any other comments?

Ask the participant to complete the SUS questionnaire.

End of interview.
