# Supplementary material for: Evaluating the usability and acceptability of a geographical information system (GIS) prototype to visualise socio-economic and public health data
Source: BMC Public Health. 2021 Nov 24;21:2151. doi: 10.1186/s12889-021-12072-1 (PMC8611402; doi:10.1186/s12889-021-12072-1)
Supplement: Supplementary file 6 — Additional file 6. Interview guide for generic interactive session [file 12889_2021_12072_MOESM6_ESM.docx]

ID:

Date:

1. What interactive aspects did you like about the model?
2. What interactive aspects did you dislike about the model?
3. What changes would you make to the model?

System Usability Scale

Strongly Strongly

disagree agree

1. I think that I would like to

use this system frequently

2. I found the system unnecessarily

complex

3. I thought the system was easy

to use

4. I think that I would need the

support of a technical person to

be able to use this system

5. I found the various functions in

this system were well integrated

6. I thought there was too much

inconsistency in this system

7. I would imagine that most people

would learn to use this system

very quickly

8. I found the system very

cumbersome to use

9. I felt very confident using the

system

10. I needed to learn a lot of

things before I could get going

with this system
